# Primary non-Hodgkin’s lymphoma of bone: poly-ostotic versus mono-ostotic subtypes

**DOI:** 10.3332/ecancer.2013.330

**Published:** 2013-07-03

**Authors:** KC Lakshmaiah, B Guruprasad, Samit Purohit, Sandesh Rao, Siddhartha Bishwas, D Lokanath

**Affiliations:** Kidwai Memorial Institute of Oncology, Dr. M.H. Marigowda Road, Bangalore 560029, Karnataka, India

**Keywords:** extranodal lymphoma, primary non-Hodgkin lymphoma of bone, chemotherapy for bone NHL

## Abstract

Primary non-Hodgkin’s lymphoma of bone (PNHLB) accounts for less than 5% of all primary bone tumours and less than 1% of all non-Hodgkin’s lymphoma. Due to its rarity, only a few retrospective studies have been published describing the prognosis and its treatment. We report our experience of 20 cases of PNHLB with their clinicopathologic correlation that were treated at our centre over a period of ten years. There were 16 cases of the mono-ostotic subtype and four cases of poly-ostotic subtype. All of these had a histological diagnosis of diffuse large B-cell lymphoma. The age of presentation was fifth to sixth decade. The mono-ostotic subtype commonly presented with the involvement of femur or humerus, while the poly-ostotic subtype commonly had paraparesis due to vertebral involvement. Cyclophosphamide, doxorubicin, vincristine, prednisone (CHOP)-based chemotherapy was given to all patients, but definitive radiotherapy was used only in the mono-ostotic subtype. At median follow-up of 38 months (range 5–96 months), event-free survival was 81% and 25% with mono-ostotic and poly-ostotic subtypes, respectively. Thus poly-ostotic PNHLB is a distinctive entity with a poor prognosis, and larger studies are needed for better management of this subtype.

## Introduction

Primary non-Hodgkin’s lymphoma of bone (PNHLB) comprises less than 5% of primary bone tumours, 5% of extranodal lymphomas, and <1% of all non-Hodgkin’s lymphoma (NHL) [1]. Being one of the rarest primary bone malignancies, PNHLB was first described by Oberling in 1928 [2]. Initially called primary reticulum cell sarcoma in 1939 by Jackson *et al *[3], the term primary lymphoma of the bone was introduced by Ivins and Dahli in 1963 [4]. The criteria for the mono-ostotic subtype were defined by Coley [5], whereas it was Ostrowski [6] who first introduced the poly-ostotic subtype into the classification. Chemotherapy remains the cornerstone of treatment, in combination with radiation and with the introduction of rituximab [7]; there has been an improvement in response rates, disease-free, and overall survival (OS).

There is a paucity of data regarding the clinical features and outcome of this disease in the Indian scenario. In this study, we have also compared the outcome of the poly-ostotic subtype with mono-ostotic subtype.

## Materials and methods

In this retrospective study, 20 cases were diagnosed and treated for PNHLB from January 2003 to December 2012 at our institute. The case records of these patients were analysed in detail for demographic profile, clinical features, imaging, treatment, and outcome. The diagnosis was established on the basis of histopathological examination and immunohistochemistry. The immunohistochemistry panel included LCA, CD20, CD10, CD15, CD30, CD3, CD79a, ALK, and EMA. The diagnosis of mono-ostotic bone lymphoma was based on the criteria of (a) a single skeletal site, with or without regional lymph node involvement, (b) positive histological diagnosis, and (c) no evidence of distant soft tissue or distant lymph node involvement. Poly-ostotic NHL was diagnosed based on the criteria of (a) multiple bone involvement but no visceral or lymph node involvement and (b) positive histological diagnosis. The staging evaluation included a chest x-ray, abdominal ultrasound, bone scan, CT scan, and bone marrow examination. In this retrospective study, all patients were staged by the Ann Arbor system and received CHOP-based chemotherapy. Due to financial constraints, both PET scan and rituximab were not used in our patients. Mono-ostotic NHL patients received local radiotherapy, and poly-ostotic NHL patients received radiotherapy when they had compressive myelopathy features. Radiotherapy for extremities was given by conventional fractionation five days per week, one fraction per day, using the parallel opposed technique via telecobalt to a total dose of around 40 Gy. The responses were classified per RECIST guideline 1.1.

The event-free survival (EFS), defined as minimum time to disease progression or relapse, was calculated using the Kaplan–Meier curve (SPSS 19, SPSS Inc., United States).

## Results

**Figure 1:**
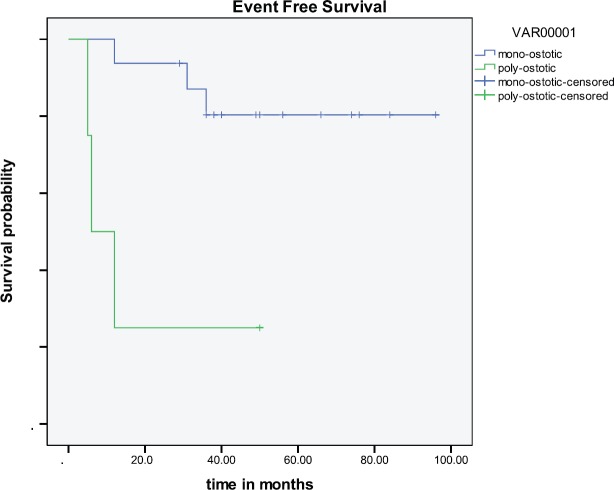
EFS (mono-ostotic versus poly-ostotic).

**Figure 2:**
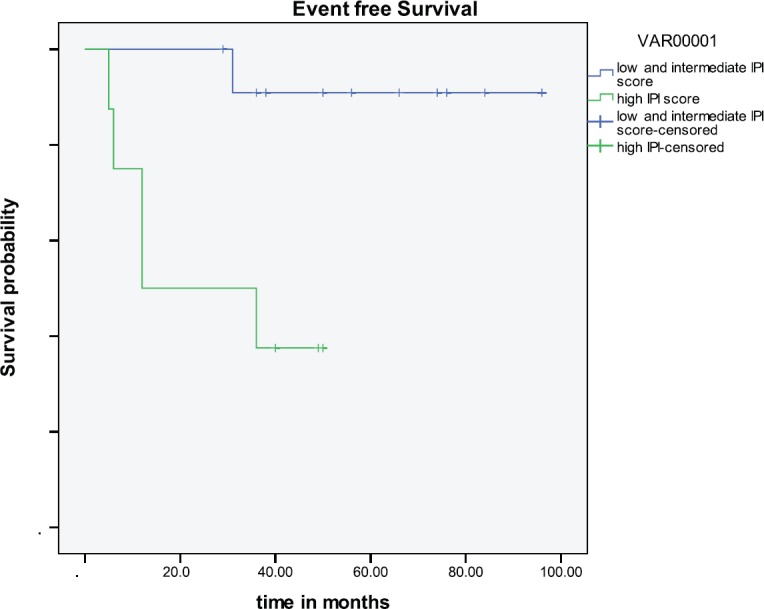
Outcome based on IPI.

**Figure 3:**
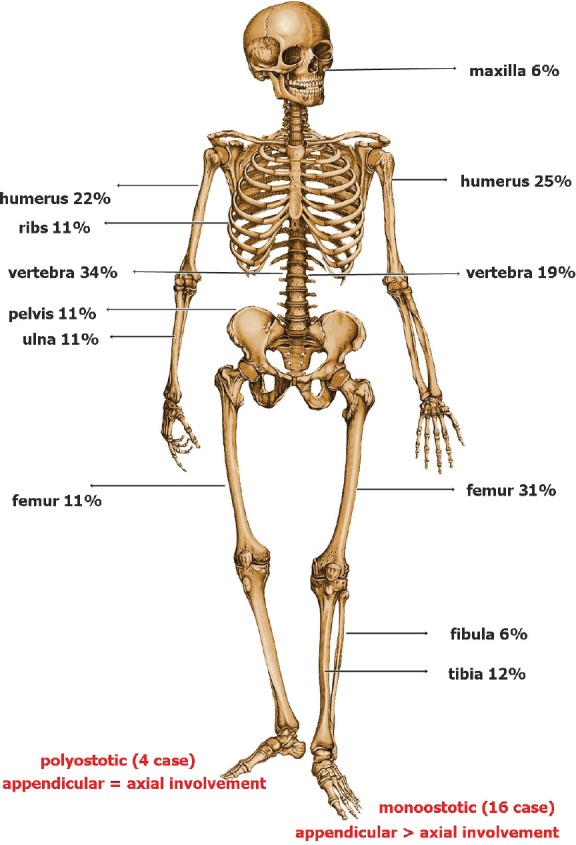
Bones involved.

As seen in Table 1, 60% of the patients were male, with a median age of 55. The mono-ostotic patients presented with a palpable mass/swelling commonly involving the femur (31%) or humerus (25%), while the poly-ostotic patients commonly had paraparesis due to vertebral involvement (Figure 3). B symptoms were present in only two cases (10%). Bone marrow did not show evidence of involvement in any patients. All cases were of diffuse large B-cell histology. The mono-ostotic patients usually presented with a low international prognostication index (IPI) score, whereas poly-ostotic patients presented with a high IPI score (Table 2). The treatment outcome is shown in Figures 1 and 2. Relapse occurred in six patients, three with the poly-ostotic subtype and three with the mono-ostotic subtype. There was no other extra nodal relapse. At the median follow-up of 38 months, the EFS for the mono-ostotic cases was 81%, and in the poly-ostotic cases, it was 25% (Figure 1). In patients with mono-ostotic disease who had complete response to initial therapy, all patients had a distant site as the pattern of relapse.

## Discussion

The aetiology of this rare bone tumour remains unknown. As primary bone lymphoma is a highly curable disease, differentiating it from other causes of lytic bone lesions such as secondaries and other primary bone tumours is important. This study highlights the need for proper diagnosis and management of PNHLB patients.

As seen in our study, patients with PNHLB commonly present with localised bone pain, soft tissue swelling, or a pathological fracture. There is a slight male preponderance, and most patients are in their fifth or sixth decade. This has been reported in other larger studies as well [7]. A review of the literature worldwide suggests that PNHLB can arise in any part of the skeleton; however, the femur is the most common site, involved in nearly one-third of cases. Other less common sites are the pelvis, humerus, skull, and tibia [8]. The involvement of the small bones in the hands and feet is rare.

As noted by Beal *et al *[9] in their study, nearly 81% of cases presented with stage I or II; this is similar to our study where the majority (80%) of the cases are limited disease per the Ann Arbor staging system.

As seen in our study and other large studies, most PNHLB are diffuse large B-cell lymphomas [10]. Rarely, less common histologies such as small-cell lymphocytic, anaplastic large cell lymphoma, and follicular lymphoma may be present.

Five-year survival has ranged from 57% [11] to 88% [9]; it was 70% in our study. The analysis of the SEER database that included 1500 patients from 1973–2005 showed that only younger age and localised disease were independent predictors of survival [10]. This is also shown in our study (Figure 1), where poly-ostotic had poor survival compared with mono-ostotic as these patients are not amenable to local therapies like surgery or radiotherapy. Other large studies have taken age and IPI scoring as prognostic markers and divided patients into three groups: (1) age < 60 with IPI 1–3, (2) age ≥ 60 with IPI 0–3, and (3) age ≥ 60 with IPI 4–5; the three groups have markedly different five-year OS of 90%, 61%, and 25%, respectively [7]. Even in our study, patients with a high IPI score had a poor outcome (*p *= 0.0181). Since there is a dearth of studies on this tumour using rituximab therapy, our study can be seen as a comparative arm for further larger studies that use rituximab.

As seen with other studies [10], poly-ostotic PNHLB is a distinctive clinical entity with a poor therapeutic outcome. Most of these are older patients who have a higher IPI score, predominantly axial skeleton involvement, and are not amenable to local therapies like surgery or radiotherapy due to multiple bone involvement. We propose limiting the name of primary bone lymphoma only to the mono-ostotic variant. Since poly-ostotic has a poor prognosis, larger studies are needed, which explore other therapeutic options and the molecular genetics of this disease.

## Figures and Tables

**Table 1.** Clinical profile.

	Mono-ostotic (16 cases)	Poly-ostotic (4 cases)
Incidence	80%	20%
Male: female	2.2:1	3:1
Mean age	54	58

**Table 2.** Stage and outcome.

	Mono-ostotic	Poly-ostotic
Stage	1E − 12 cases	IV − 4 cases
	IIE − 4 cases	
Low IPI score	8 (50%)	
Intermediate IPI score	4 (25%)	
High IPI score	4 (25%)	4 (100%)
Chemotherapy+ radiotherapy	All cases received definitive RT	RT given for spinal compression
EFS	81%	25%
Pattern of failure	Distant failure	Local relapse

EFS: event-free survival.

## References

[1] Freeman C, Berg JW, Cutler SJ (1972). Occurrence and prognosis of extranodal lymphomas. Cancer.

[2] Oberling C (1928). Les reticulosarcomes et les reticuloendotheliosarcomes de la moelle osseuse (sarcoma d’Ewing). Bul Assoc Fr Etud Cancer (Paris).

[3] Parker F, Jackson H (1939). Primary reticulum cell sarcoma of bone. Surg Gynecol Obst.

[4] Ivins JC, Dahlin DC (1963). Malignant lymphoma (reticulum cell sarcoma) of bone. Mayo Clin Proc.

[5] Coley BL, Higginbotham NL, Groesbeck HP (1950). Primary reticulum cell sarcoma of bone: summary of 37 cases. Radiology.

[6] Ostrowski ML, Unni KK, Banks PM (1986). Malignant lymphoma of bone. Cancer.

[7] Ramadan KM, Shenkier T, Sehn LH (2007). A clinicopathological retrospective study of 131 patients with primary bone lymphoma: A population-based study of successively treated cohorts from the British Columbia Cancer Agency. Ann Oncol.

[8] Salter M, Sollaccio RJ, Bernreuter WK (1989). Primary lymphoma of bone: The use of MRI in pretreatment evaluation. Am J Clin Onco.

[9] Beal K, Allen L, Yahalom J (2006). Primary bone lymphoma: treatment results and prognostic factors with long-term follow-up of 82 patients. Cancer.

[10] Jawad MU, Schneiderbauer MM, Min ES, Cheung MC, Koniaris LG, Scully SP (2010). Primary lymphoma of bone in adult patients. Cancer.

[11] Lewis VO, Primus G, Anastasi J (2003). Oncologic outcomes of primary lymphoma of bone in adults. Clin Orthop Relat Res.

[12] Fairbanks RK, Bonner JA, Inwards CY, Strickler JG, Habermann TM, Unni KK, Su J (1994). Treatment of stage IE primary lymphoma of bone. Int J Radiat Oncol Biol Phys.

